# Ultrasensitive enzyme-free fluorescent detection of VEGF_165_ based on target-triggered hybridization chain reaction amplification†

**DOI:** 10.1039/c8ra04721a

**Published:** 2018-07-19

**Authors:** Qingzhen Zhou, Hongxia Yan, Fengying Ran, Jianjun Cao, Long Chen, Bing Shang, Hao Chen, Jian Wei, Qinhua Chen

**Affiliations:** Affiliated Dongfeng Hospital, Hubei University of Medicine Hubei Shiyan 442008 China cqh77@163.com +86 0719-8272283; Department of Radiotherapy, Hubei Cancer Hospital 116 South Zuodaoquan Road Wuhan 430074 China

## Abstract

Sensitive detection of vascular endothelial growth factor (VEGF_165_) is important for early cancer disease diagnosis in the clinic. A sensitive fluorescent sensing platform for VEGF_165_ detection is developed in this work. It is based on a target-triggered hybridization chain reaction (HCR) and graphene oxide (GO) selective fluorescence quenching. In this assay, in the presence of the VEGF_165_, the hairpin structure of Hp opens up and the initiation sequence will be exposed to Hp1 to open its hairpin structure. Then the opened Hp1 hybridizes with Hp2 to expose the complementary sequence of Hp1 which hybridizes with Hp1 again by HCR. Thus HCR would be initiated, generating super-long dsDNA. After the HCR, the double strands of the HCR product cannot be adsorbed on the GO surface. As a result, the HCR product gives a strong fluorescence signal which is dependent on the concentration of VEGF_165_. By using VEGF_165_ as a model analyte, the assay provides a highly sensitive fluorescence detection method for VEGF_165_ with a detection limit down to 20 pg mL^−1^. The proposed aptasensing strategy based on target-triggered HCR amplification can thus be realized. It was successfully applied to the determination of VEGF_165_ in spiked human serum, urine and saliva. Therefore, it can easily have wide applications in the diagnosis of vital diseases.

## Abstract 2

Sensitive detection of vascular endothelial growth factor (VEGF_165_) is important for early cancer disease diagnosis in the clinic.

## Graphical abstract

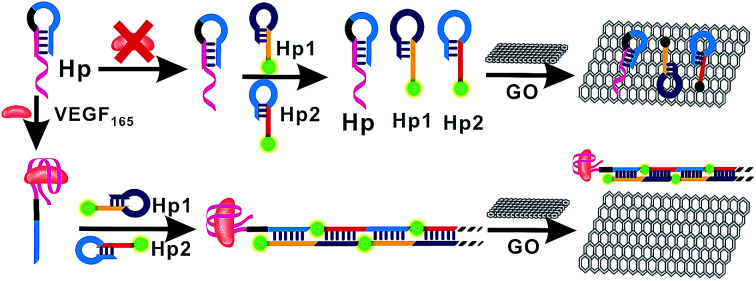

## 1. Introduction

Cancer has become one of the greatest causes of human death worldwide,^1^ and so early diagnosis of cancer is particularly important in reducing cancer mortality. Nowadays, techniques for the clinical diagnosis of cancer depend primarily on the biopsy of diseased cells or tissues and imaging equipment.^2^ However, these methods are limited by their low sensitivity in the diagnosis of cancer at an early stage. Biomarkers, including cells, nucleic acids, and proteins, could be used as indicators for the evaluation of either normal or pathological processes.^3,4^ The vascular endothelial growth factor (VEGF_165_) is a significant signal protein stimulating vasculogenesis and is secreted by both endothelial and tumor cells.^5^ It plays a central role in the regulation of the angiogenesis process.^6^ However, VEGF_165_ is usually over-expressed in variants of breast and lung cancer and may be a possible predictor of cancers.^7–11^ VEGF_165_ may be an important biomarker for early cancer disease diagnosis in clinical settings.^12,13^ Thus, the rapid, selective and sensitive detection of VEGF_165_ is important for disease diagnosis and subsequent therapy monitoring.

To date, various VEGF_165_ detection techniques have been reported, including ELISA assays,^14–16^ immunohistochemistry, optical methods,^17^ and radioimmunoassay. However, these methods are mainly time-consuming, labor-intensive, and complex and involve expensive reagents. Several novel biosensor methods have also been reported for the detection of VEGF_165_, such as the use of aptamers. Aptamers are short oligonucleotide sequences (ssDNA or RNA), and could be screened *in vitro* and synthesized with the systematic evolution of ligands.^18^ The aptamers have high affinity, specificity, and stability, and simple synthesis, easy labeling and wide applicability compared to traditional molecular recognition systems.^19^ Therefore, they are regarded as an alternative to antibody-based methods. Up to now, a variety of aptamer-based analytical strategies for the detection of VEGF_165_ have been reported, such as differential pulse voltammetry (DPV),^20,21^ electrochemical impedance spectroscopy (EIS),^11^ photoelectrochemical (PEC),^22^ electrochemiluminescence (ECL),^23^ chemiluminescence (CL),^24,25^ and time-resolved fluorescence (TR-FL).^26^ These methods have good specificity and stability compared to traditional approaches.^27,28^ However, they lack a signal amplification strategy, resulting in low sensitivity, a narrow detection range and high error rate. For these reasons, a highly sensitive and selective method with a wide quantitative dynamic range is essential for VEGF_165_ detection.

Recently, aptamer-based amplification assays, such as the rolling chain reaction (RCA),^29^ polymerase chain reaction assay (PCR),^30^ ligase chain reaction (LCR),^31^ and nanoparticle-assisted amplification^32^ have aroused more attention due to their high sensitivity and wide quantitative dynamic range. However, these methods are highly enzyme-dependent, costly and sensitive to reaction conditions. In addition, the reaction conditions, such as the pH, temperature and buffer media, must be precisely controlled in order to guarantee the activity of the enzymes.^33^

As far as we know, the hybridization chain reaction (HCR), one of the most attractive enzyme-free amplification methods, shows great potential in nucleic acid detection.^34,35^ In an HCR system, two DNA hairpin probes Hp1 and Hp2 can coexist stably in solution unless triggered by a primer sequence.^36,37^ When the target was introduced, the hairpin probes changed, initiating a cascade of hybridization events.^38^ Therefore, HCR has been regarded as an ideal choice in DNA-based signal amplification for protein detection.^39^ Graphene oxide (GO) has gained more and more attention due to its large surface area, good water dispersibility and biocompatibility.^40,41^ Moreover, GO can strongly adsorb single-stranded nucleic acids, but hardly interacts with rigid double-stranded nucleic acids. At the same time, the fluorescence signal of the substances adsorbed by GO can be quenched. Therefore, GO can be used as an ideal nanomaterial in fluorescent biosensors.^42,43^

Herein, we designed a highly sensitive enzyme-free and rapid aptasensing platform for the detection of VEGF_165_ based on the HCR. In this assay, in the presence of the VEGF_165_, the hairpin structure of Hp opens up and the initiation sequence will be exposed to Hp1 to open its hairpin structure by a toehold-mediating strand displacement reaction. Once the hairpin of the first Hp1 opens up, it triggers a cascade reaction, resulting in opening up the hairpin of Hp2, and consequently Hp1, Hp2, Hp1, Hp2 …. Thus the HCR would be initiated, generating super-long dsDNA. The double strands of the HCR product cannot be adsorbed on the GO surface.^44^ As a result, the HCR product gives a strong fluorescence signal dependent on the concentration of VEGF_165_. More importantly, this simple and rapid amplification strategy can be completed in a short time without expensive enzymes, and provides a novel approach for detection of VEGF_165_. Furthermore, it has been successfully applied to the detection of VEGF_165_ in various spiked biological samples, with recovery in the range of 99.7–107.2%. Therefore, one may believe that this sensing system possesses great potential for detection of proteins, biological molecules research and clinical diagnosis.

## 2. Experimental section

### 2.1. Reagents and materials

The EpCAM, PSA, BSA and VEGF_165_ were purchased from Cusabio Biotech Co. Ltd. All of the reagents were diluted to the required concentration with working buffer (20 mM Tris–HCl, 100 mM NaCl, pH 7.4) before use. All of the oligonucleotides used in this work were synthesized and purified using HPLC by Sangon Biotechnology Co. Ltd. (Shanghai, China, https://www.sangon.com), and their sequences are shown in Table S1.† GO was purchased from XFNANO Co. Ltd. (Nanjing, China, https://www.xfnano.com). The other reagents employed were of analytical grade and used without further purification. Healthy human serum, urine and saliva were provided by Affiliated Dongfeng Hospital, Hubei University of Medicine, and approved by the Hospital's Ethics Committee. Ultrapure water obtained from a Millipore water purification system (18.2 MΩ cm resistivity, Milli-Q Direct 8) was used in all runs.

### 2.2. VEGF_165_ sensing procedure

Firstly, a mixture of Hp as well as Hp1 and Hp2 strands were separately heated at 90 °C for 5 min, respectively, followed by slow cooling down to room temperature. Next, different concentrations of VEGF_165_ were incubated with the Hp solution in working buffer (20 mM Tris–HCl, 100 mM NaCl, pH 7.4) for 30 min at 37 °C. This was followed by the addition of Hp1 and Hp2 and incubation at 37 °C. Finally, the GO was added and incubated with the reaction solution for 5 min. Then the solution was diluted to 1 mL, and the fluorescence intensity of the solution was measured.

### 2.3. Fluorescence measurements

The fluorescence detection of the mixtures was carried out on a Hitachi F-4600 spectrophotometer (Hitachi Co. Ltd., Japan, https://www.hitachi.co.jp) equipped with a xenon lamp excitation source at room temperature. The excitation was set at 495 nm and the emission spectra were collected from 510 nm to 600 nm. The slits of the excitation and emission were both set at 5 nm. In control experiments, the measurement process was the same as the above except for the addition of VEGF_165_. Unless otherwise noted, each fluorescence measurement was repeated three times, and the standard deviation was plotted as the error bar. To evaluate the effects of potential interfering substances on detection, three different relevant compounds, EpCAM, BSA and PSA at a concentration of 100 ng mL^−1^, were spiked, respectively, and the measurements were performed under the same conditions. A quantitative assay of VEGF_165_ was realized by using the fluorescence intensity. *F*_1_ and *F*_0_ are the fluorescence intensities at 519 nm in the presence and absence of VEGF_165_, respectively.

## 3. Results and discussion

### 3.1. Principle of design

**Fig. 1:**
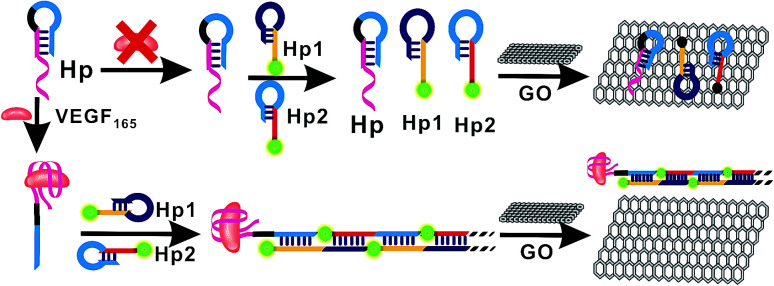
Schematic illustration of the fluorescence assay detection of VEGF_165_ based on target-triggered hybridization chain reaction amplification.

In the present study, the principle of enzyme-free fluorescent detection of VEGF_165_ based on HCR is illustrated in Fig. 1. The detection method was composed of three DNA probes, assistant DNA probe (Hp), hairpin probe 1 (Hp1) and hairpin probe 2 (Hp2), and VEGF_165_ was adopted as the target protein. Hp sequences include a VEGF_165_ aptamer (in red), extended-DNA (E-DNA, in blue) which is complementary to part of Hp1 on the 5′ terminal (in yellow), and connecting 10 successive adenine bases (in italics) between them to reduce any possible steric hindrance effect, without interaction with other DNA sequences or molecules. Among these, the Hp1 on the 5′ terminal was labeled with an FAM, and the Hp2 on the 3′ terminal was labeled with an FAM. In the absence of VEGF_165_, the HCR process cannot be triggered. This results in the coexistence of Hp, Hp1 and Hp2 in solution, which can be adsorbed on the GO surface through their sticky ends and loops, resulting in a very weak fluorescence signal. In the presence of VEGF_165_, the aptamer sequences in Hp could be recognized by VEGF_165_ to form a VEGF_165_–aptamer complex, and then the E-DNA (in blue) originally caged in Hp was liberated. The liberated E-DNA hybridizes with the sticky end of Hp1 (the yellow region) and opens the hairpin DNA through the toehold-aided strand displacement reaction. The newly released sticky sequence of Hp1 (the dark blue region) further hybridizes with the sticky end of Hp2 (the red region), to open the Hp2 hairpin DNA and expose a new sticky end on Hp2 (the blue region) being the same as E-DNA. The continuous strand displacement reactions generate a long chain of Hp leading to complexes of Hp1 and Hp2. Thus the HCR would be initiated, generating super-long dsDNA. After the HCR reaction, the GO was added into the solutions, and the free Hp1 and Hp2 were closely adsorbed onto the GO surface *via* π–π stacking. However, the HCR product cannot be adsorbed on the GO surface,^45,46^ realizing a strong fluorescence signal.

### 3.2. Feasibility analysis of fluorescence VEGF_165_ detection

**Fig. 2:**
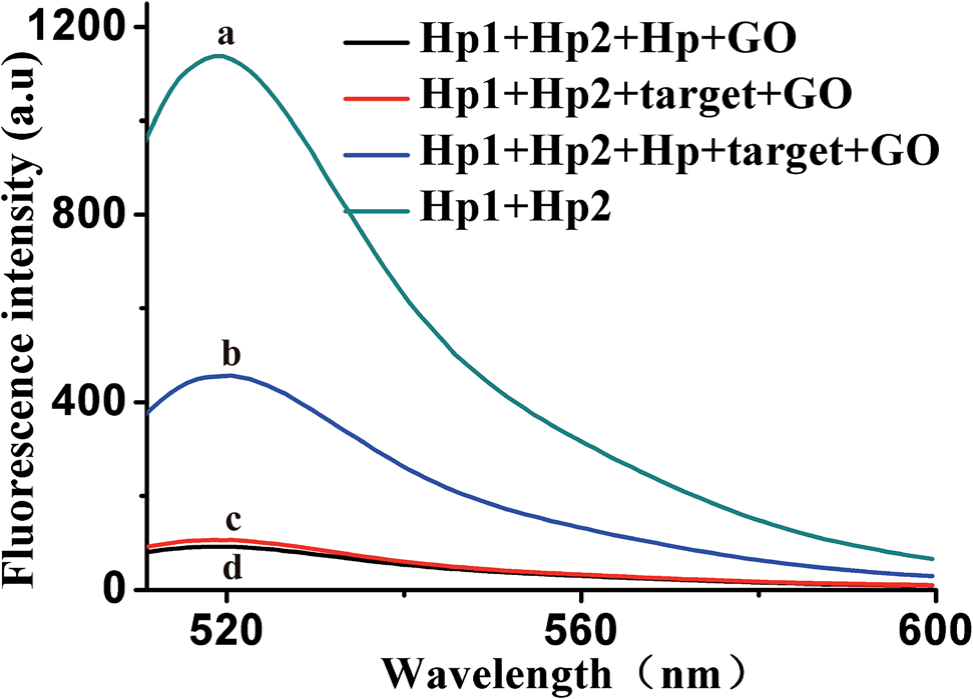
The typical fluorescent emission spectra of different mixtures, biosensing platform from curve a to d: (a) Hp1 + Hp2; (b) Hp1 + Hp2 + Hp + target + GO; (c) Hp1 + Hp2 + target + GO; (d) Hp1 + Hp2 + Hp + GO. The concentrations of Hp1, Hp2, Hp, GO and target (VEGF_165_) were 30 nM, 30 nM, 5 nM, 20 μg mL^−1^ and 10 ng mL^−1^, respectively.

To further verify the feasibility of the HCR/GO fluorescence signal amplification strategy, Fig. 2 shows the fluorescence emission spectra under different conditions. The fluorescence signal produced by a mixed solution of Hp1 and Hp2 (curve a) is relatively strong in the absence of GO. After adding the target (curve c) or Hp (curve d), the fluorescence intensity was relatively weak in the presence of GO. The reason may be that, when target and Hp were added alone, the HCR reaction could not occur, giving the results that appear in the graph. However, when 10 ng mL^−1^ of VEGF_165_ was added into a mixed solution of Hp1, Hp2 and Hp, a significant enhancement in the fluorescence intensity was observed (curve b) in the presence of GO, indicating that VEGF_165_ was specifically recognized and triggered the HCR events. These results confirm the feasibility of the proposed fluorescence biosensor for VEGF_165_ detection by our design.

### 3.3. Optimization of reaction conditions

**Fig. 3:**
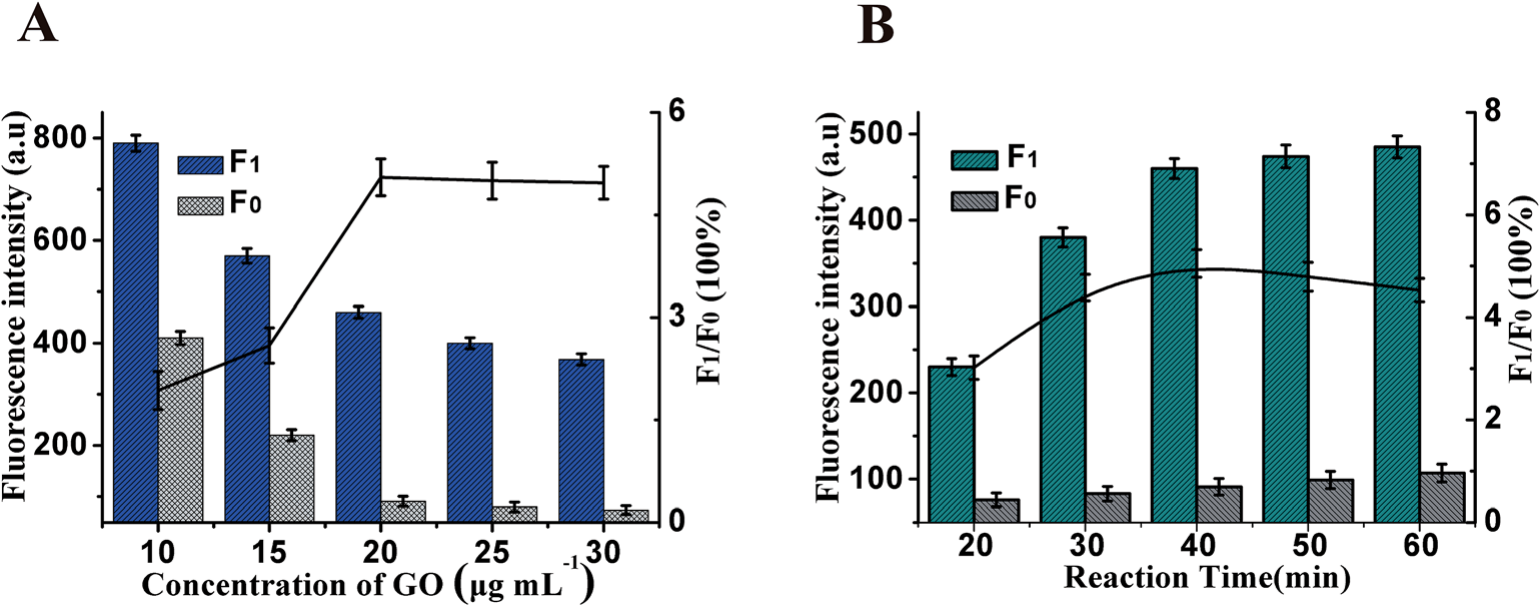
(A) The effect of GO concentration on the fluorescence response of this method. (B) The fluorescence responses of optimization of HCR time. Light gray columns: control experiments; dark blue and green columns: with 10 ng mL^−1^ of target; both Hp1 and Hp2 are 30 nM. The black lines represent the *F*_1_/*F*_0_ at different conditions, where *F*_1_ and *F*_0_ are the fluorescence intensities in the presence and absence of VEGF_165_, respectively. Error bars: SD, *n* = 3.

Whether the fluorescence of Hp1 and Hp2 could be effectively quenched by GO is vital for the design of the HCR/GO assay, which is firstly explored in this paper. The fluorescence change is related to the concentration of GO shown in Fig. 3A. The maximum *F*_1_/*F*_0_ value was observed when the concentration of GO was 20 μg mL^−1^. When the concentration of GO is increased, however, the *F*_1_/*F*_0_ value obviously decreases along with the increasing GO concentration. Therefore, 20 μg mL^−1^ is selected as the optimum GO concentration. At the same time, the reaction time of the HCR is another important factor affecting fluorescence intensity. As shown in Fig. 3B, maximum *F*_1_/*F*_0_ values are observed when the reaction time is 40 min; therefore, 40 min of HCR time was selected for the rest of the experiments.

Besides the above conditions, the pH and HCR reaction temperature may also affect the sensitivity. As shown in Fig. S1A,† it can be seen that the fluorescence intensity initially increased and then decreased with an increase in pH. The maximum *F*_1_/*F*_0_ values are observed when the pH is 7.4; therefore, the best pH was pH 7.4. Fig. S1B† shows that the incubation temperature could obviously affect the sensitivity, and the *F*_1_/*F*_0_ value reached a maximum when the incubation temperature was 37 °C and then decreased gradually; thus, an HCR reaction temperature of 37 °C was selected in the experimental design.

To obtain high sensitivity, the concentrations of Hp1, Hp2 and Hp were also investigated. As shown in Fig. S2A,† the concentration of Hp1 was selected in the following experiments; the *F*_1_/*F*_0_ value reached a maximum when the concentration of Hp1 was 30 nM. Therefore, a concentration of 30 nM was selected as the optimal concentration of Hp1, and the concentration of Hp2 is identical to that of Hp1. As shown in Fig. S2B,† the concentration of Hp was selected in the following experiments. The maximum *F*_1_/*F*_0_ value was observed when the concentration of Hp was 5 nM; thus, 5 nM was confirmed as the optimum reaction concentration of Hp.

### 3.4. Sensitivity and specificity for VEGF_165_ detection

**Fig. 4:**
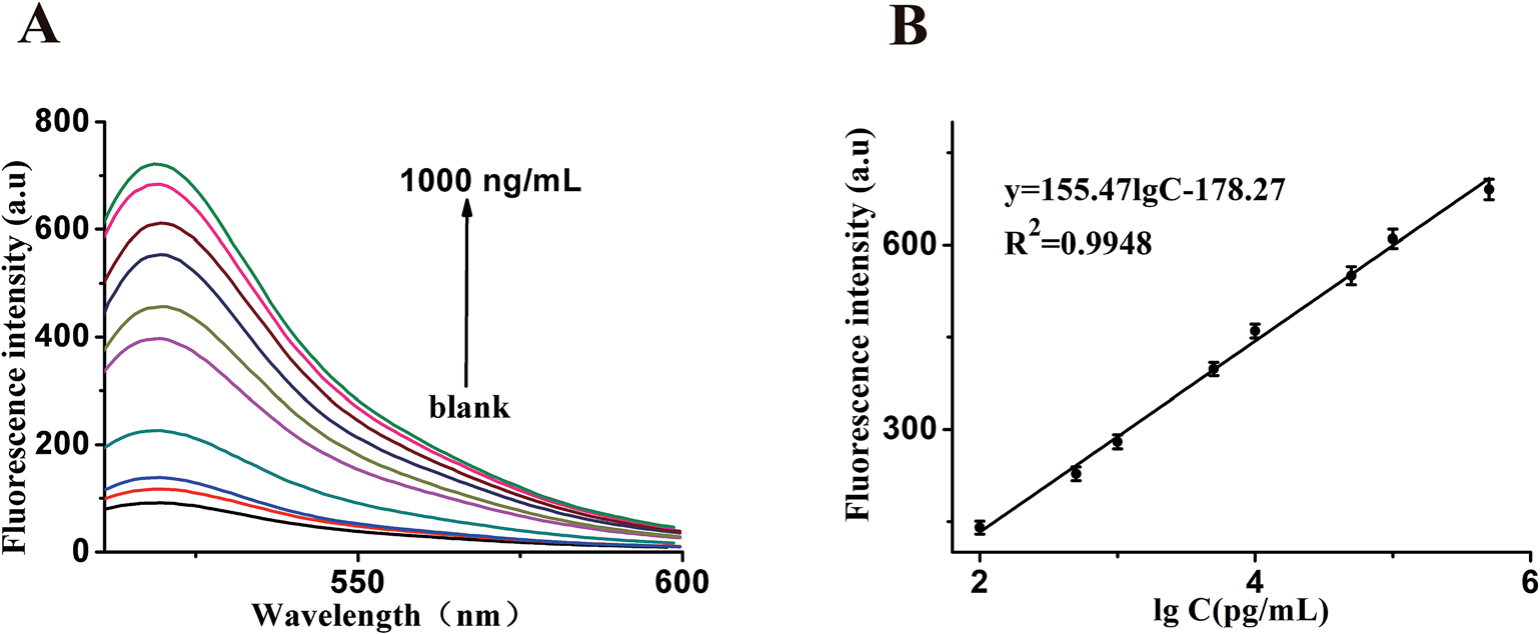
(A) Fluorescence emission spectra of the biosensor in the presence of VEGF_165_ with different concentrations: from bottom to top: 0 to 1000 ng mL^−1^. (B) The relationship curve of fluorescence intensity as a function of VEGF_165_ concentration. It shows the relationship between fluorescence intensity and VEGF_165_ concentration, under both experimental conditions: Hp1, 30 nM; Hp2, 30 nM; Hp, 5 nM; GO, 20 μg mL^−1^ and an emission wavelength of 519 nm. Error bars: SD, *n* = 3.

In order to make better use of this designed strategy, the sensitivity of the sensor for the detection of VEGF_165_ was investigated. As shown in Fig. 4A, in an analysis of different quantities of target, we found that the fluorescence intensity was obviously enhanced with an increased quantity of target. Fig. 4B shows that a good linear correlation between the fluorescence intensity and the logarithm of VEGF_165_ concentration ranging from 0.1 ng mL^−1^ to 500 ng mL^−1^ was obtained. The regression equation was expressed as *y* = 155.471 log *c* − 178.27 (*R*^2^ = 0.9948), where log *c* is the logarithm of VEGF_165_ concentration (pg mL^−1^). According to the 3σ rule, the detection limit was calculated to be 20 pg mL^−1^. In comparison, such a low detection limit was comparable to or even higher than those reported in the literature and listed in Table 1.

**Table 1:** A comparison of the designed method for the detection of VEGF_165_ with other reported biosensors

Analytical method	Detection limit	Linear range	Ref.
Differential pulse voltammetry	26.7 pg mL^−1^	0.1–12.2 ng mL^−1^	20
Electrochemical impedance spectroscopy	1.0 pg mL^−1^	10.0–300.0 pg mL^−1^	11
Photoelectrochemical	1.1 pg mL^−1^	0.003–382 ng mL^−1^	22
Electrochemiluminescence	7.6 pg mL^−1^	0.04–764 pg mL^−1^	23
Differential pulse voltammetry	12.2 pg mL^−1^	0.04–4.6 ng mL^−1^	21
Chemiluminescence	1.0 ng mL^−1^	1–20 ng mL^−1^	24
Time-resolved fluorescence	3.1 ng mL^−1^	0.4–611.2 ng mL^−1^	26
Colorimetric biosensor	65 pg mL^−1^	10–430 ng mL^−1^	25
Fluorescent biosensor	20 pg mL^−1^	0.1–500 ng mL^−1^	This method

**Fig. 5:**
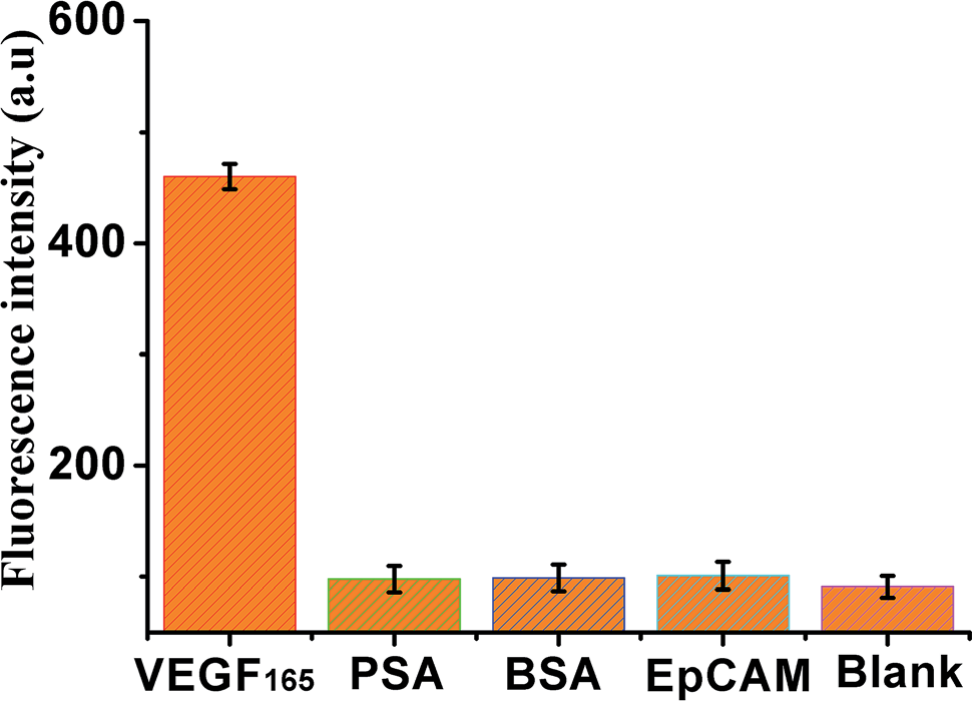
Fluorescence intensity (at an emission wavelength of 519 nm) of the sensor in the presence of VEGF_165_ (10 ng mL^−1^), PSA (100 ng mL^−1^), BSA (100 ng mL^−1^), EpCAM (100 ng mL^−1^) and black, respectively. Error bars: SD, *n* = 3.

In addition, the specificity of the sensor was also investigated by adding four kinds of different control proteins, VEGF_165_, PSA, BSA and EpCAM, as shown in Fig. 5, weak fluorescence was exhibited in the presence of PSA, BSA, or EpCAM, compared with the blank control. However, a significant increase in fluorescence signal was observed in the presence of VEGF_165_, indicating that this method exhibited good specificity for VEGF_165_ detection.

### 3.5. Determination of VEGF_165_ in real samples

**Fig. 6:**
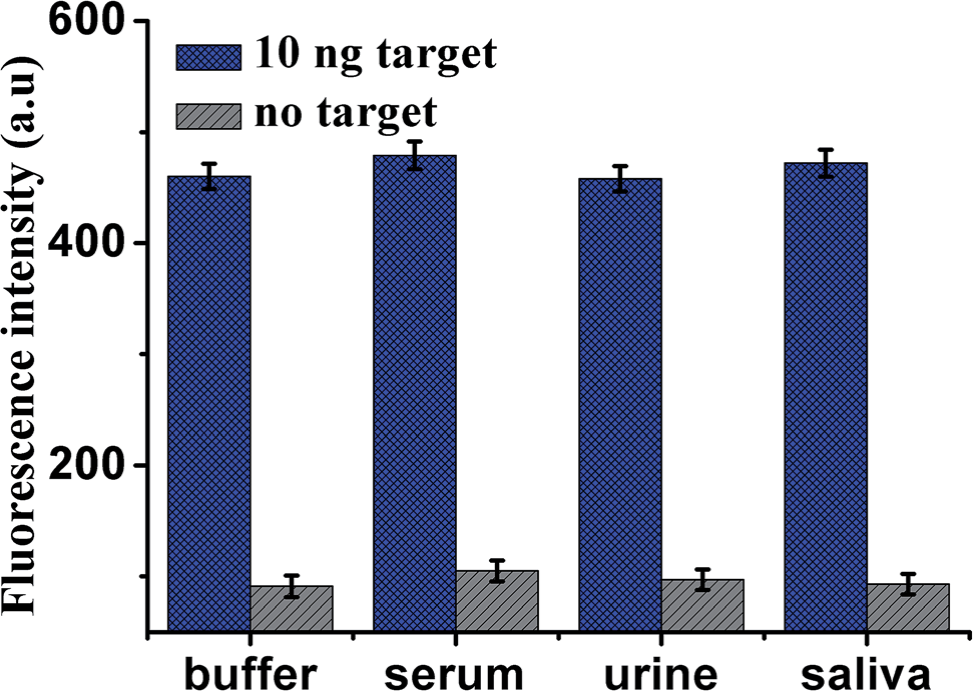
Fluorescence intensity of the sensor for the detection of VEGF_165_ in buffer and blank biological samples (human serum, urine and saliva, respectively). Error bars: SD, *n* = 3.

To assess the application of this proposed design in biological samples (all spiked samples were diluted to 10%), human serum, urine and saliva, respectively, were employed as a complex matrix. Before the measurements, the samples were prepared by adding VEGF_165_ into blank biological samples including human serum, urine and saliva, respectively. As shown in Fig. 6, the enhancement in fluorescence intensity is observed in various biological samples in the presence of 10 ng mL^−1^ VEGF_165_, compared with unspiked biological samples. The results indicate that use of the proposed sensor to analyze VEGF_165_ in biological samples was feasible. The recoveries of VEGF_165_ spiked to different matrices are listed in Table 2. The recoveries for the spiked VEGF_165_ in human serum, saliva and urine were in the range of 107.2–99.7%, and the RSD (%) were 10.8%, 10.3%, 9.9% at 10 ng mL^−1^ of VEGF_165_, respectively. This indicates an acceptable precision and reproducibility of the present approach for detecting VEGF_165_ in real samples (*n* = 3).

**Table 2:** Recoveries of VEGF_165_ spiked to different matrices

Matrix	VEGF_165_ added/ng	VEGF_165_ found/ng	Recovery (100%)	RSD (%)
Buffer	10.0	9.98^a^	99.8	9.6
Serum	10.0	10.72^a^	107.2	10.8
Saliva	10.0	10.26^a^	102.6	10.3
Urine	10.0	9.97^a^	99.7	9.9

a Mean values of three measurements.

## 4. Conclusions

In conclusion, a novel and enzyme-free highly sensitive assay of fluorescent signal amplification was successfully developed for detecting VEGF_165_. In this design, because of the specific recognition of aptamer for VEGF_165_ and signal amplification by HCR, selectivity and sensitivity for VEGF_165_ were achieved. Besides, the HCR reaction has strong environmental adaptability due to there being no participation of enzymes. Under optimal conditions, the detection limit was as low as 20 pg mL^−1^, and a linearity ranging from 0.1 ng mL^−1^ to 500 ng mL^−1^ was obtained. More importantly, this biosensor was successfully applied for the detection of VEGF_165_ in various spiked biological samples, and provides a novel approach for the detection of VEGF_165_ compared to other reported biosensors. One may believe that it could be a potential method for proteomics and clinical analysis.

## Conflicts of interest

There are no conflicts to declare.

## Supplementary Material

RA-008-C8RA04721A-s001
