# Supplementary material for: Ultrasensitive enzyme-free fluorescent detection of VEGF165 based on target-triggered hybridization chain reaction amplification
Source: RSC Adv. 2018 Jul 19;8(46):25955–60. doi: 10.1039/c8ra04721a (PMC9086580; doi:10.1039/c8ra04721a)
Supplement: RA-008-C8RA04721A-s001 [file RA-008-C8RA04721A-s001.pdf]

Electronic Supplementary Material (ESI) for RSC Advances.
This journal is © The Royal Society of Chemistry 2018

Supporting Information

Table S1. Sequences of the oligonucleotides used in this work[a]

Name	Sequence (5′ – 3′)
Hp	CTGGAAGACGGAAGCCAAACCA*AAAAAAAAAA***CCGTCTTCCAGACAAGA GTGCAGGG**
Hp1	FAM—TGGTTTGGCTTCCGTCTTCCAGTAGTAGAGGGTCTGGAAGACGG
Hp2	CTGGAAGACGGAAGCCAAACCACCGTCTTCCAGACCCTCTACTA—FAM

[a] In Hp, the boldface letters red represent the $VEGF_{165}$ recognition sequences, and the letters in italic represent the sequences of the spacer DNA between the $VEGF_{165}$ aptamer and E-DNA. The Hp1 on the 5′ terminal was labeled with a fluorophore carboxy fluorescein (FAM), and the Hp2 on the 3′ terminal was labeled with a FAM. The blue letters in Hp, Hp1 and Hp2 represent the sequences complementary to each other, respectively, and the black letters in Hp1 and Hp2 represent the sequences complementary to each other.

Optimization of pH and reaction temperature

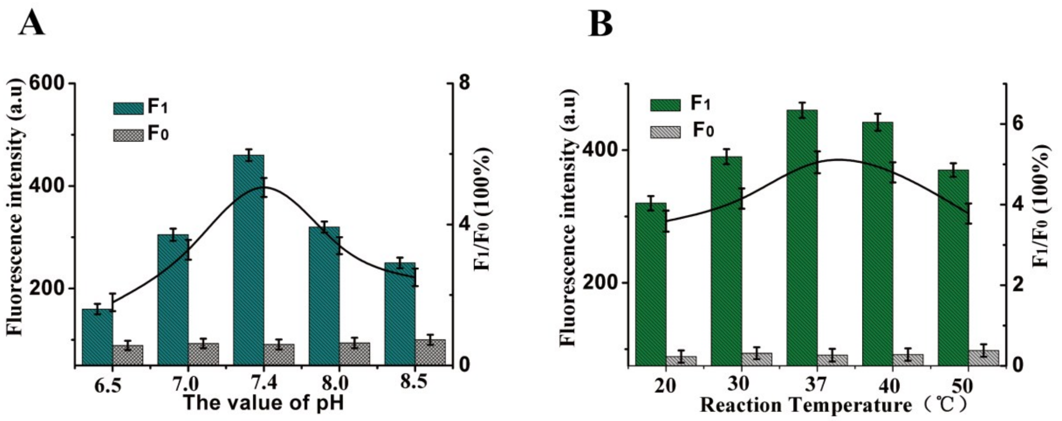

Fig. S1. The effect of pH and reaction temperature on the fluorescence intensity at the emission wavelength of 519 nm. The concentrations of Hp1, Hp2, Hp, GO and target（$VEGF_{165}$）were 30nM, 30nM, 5nM, 20 μg·mL^{-1} and 10 ng·mL^{-1}, respectively. Error bars: SD, n=3.

Optimization of Hp1 and Hp

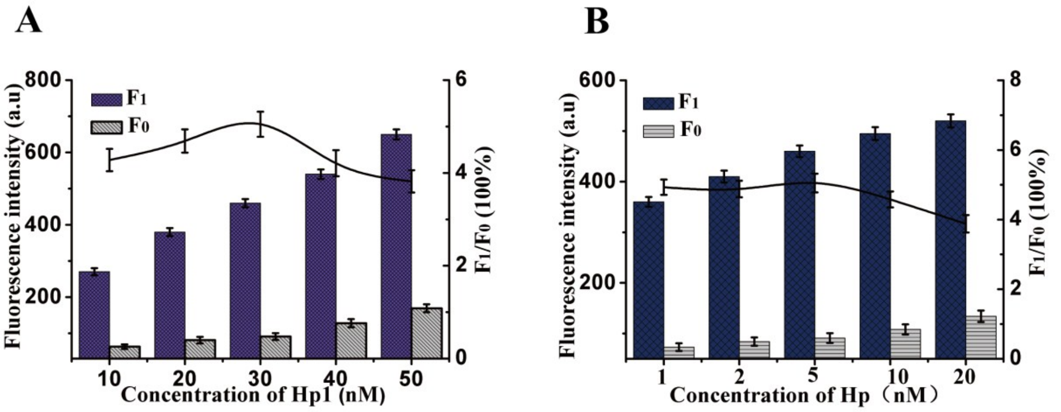

Fig. S2. The effect of Hp1 and Hp on the fluorescence intensity at the emission wavelength of 519 nm. The concentrations of GO and target（$VEGF_{165}$）were 20 μg·mL^{-1} and 10 ng·mL^{-1}, respectively. The concentrations of Hp2 are identical Hp1, Error bars: SD, n=3.
